# Characterization of Inflammatory Response in Acute-on-Chronic Liver Failure and Relationship with Prognosis

**DOI:** 10.1038/srep32341

**Published:** 2016-08-31

**Authors:** Cristina Solé, Elsa Solà, Manuel Morales-Ruiz, Guerau Fernàndez, Patricia Huelin, Isabel Graupera, Rebeca Moreira, Gloria de Prada, Xavier Ariza, Elisa Pose, Núria Fabrellas, Susana G. Kalko, Wladimiro Jiménez, Pere Ginès

**Affiliations:** 1 Liver Unit, Hospital Clínic de Barcelona, University of Barcelona, Barcelona, Spain; 2 Institut d’Investigacions Biomèdiques August Pi i Sunyer (IDIBAPS), Barcelona, Spain; 3 Centro de Investigación Biomédica en Red de Enfermedades Hepáticas y Digestivas (CIBEReHD), Barcelona, Spain; 4 Biochemistry and Molecular Genetics Department, Hospital Clínic de Barcelona, Barcelona, Spain; 5 Bioinformatics Core Facility, IDIBAPS-CEK, Hospital Clínic, University de Barcelona, Spain; 6 Facultat de Medicina i Ciències de la Salut, University of Barcelona, Barcelona, Spain

## Abstract

ACLF is characterized by a systemic inflammatory response, but the cytokines involved in this process have not been well studied. The aim of this study was to characterize the systemic inflammatory response in patients with cirrhosis and ACLF and its relationship with prognosis. Fifty-five patients with cirrhosis, 26 with ACLF, were studied prospectively. Systemic inflammatory response was analyzed by measuring a large array of plasma cytokines by using a multiplex kit. A principal component analysis show noticeable differences between ACLF and decompensated cirrhosis without ACLF. Patients with ACLF had significant abnormal levels of 12 cytokines compared to those without ACLF, including: VCAM-1, VEGF-A, Fractalkine, MIP-1α, Eotaxin, IP-10, RANTES, GM-CSF, IL-1β, IL-2, ICAM-1, and MCP-1. Cytokines showing the most marked relationship with ACLF were VCAM-1 and VEGF-A (AUCROC 0.77; p = 0.001). There was a significant relationship between some of inflammatory mediators and 3-month mortality, particularly VCAM-1, ICAM-1, and GM-CSF (AUCROC>0.7; p < 0.05). Functional Enrichment Analysis showed that inflammatory markers differentially expressed in ACLF patients were enriched in leukocyte migration, particularly monocytes and macrophages, and chemotaxis pathways. In conclusion, ACLF is characterized by a marked inflammatory reaction with activation of mediators of adhesion and migration of leukocytes. The intensity of the inflammatory reaction correlates with prognosis.

Liver cirrhosis is a chronic disease characterized by relentless deposition of collagen and disruption of the normal liver architecture that causes progressive portal hypertension and liver failure that eventually leads to complications and death unless liver transplantation is performed^1^. There is increasing evidence supporting the existence of a systemic inflammatory reaction in cirrhosis that contributes to complications and disease progression^2^,^3^. This systemic inflammatory reaction is likely initiated by translocation of bacteria or bacterial products from the intestinal lumen to the mesenteric lymph nodes and then reaching the systemic circulation. This leads to increased levels of pathogen-associated molecular patterns (PAMPs) that stimulate pattern recognition receptors (PRRs), expressed on innate immune cells. Moreover, the generation of damage-associated molecular patterns (DAMPs) from the diseased liver may also stimulate immune cells. Once stimulated, PRRs induce a transcriptional response leading to synthesis of a number of pro and anti-inflammatory cytokines, chemokines, cell adhesion molecules responsible for an adaptive immune response^4^,^5^. It has been shown that treatments that reduce bacterial translocation reduce the intensity of the immune response, while a number of complications, particularly bacterial infections are associated with an increased intensity of the immune reaction^6^,^7^. It is currently believed that such “chronic” inflammatory response may lead to a paralysis of the immune system, which in turn may be pathogenically related to the high frequency of severe infections that occur in patients with cirrhosis^8^,^9^.

There is growing interest among clinicians and researchers about acute-on-chronic liver failure (ACLF), a syndrome that occurs in patients with chronic liver diseases, particularly cirrhosis, which is characterized by development of failure of different organs and systems and high mortality rate^10^,^11^. The hypothesis has been raised that ACLF is associated with a remarkable inflammatory state that contributes to the pathogenesis and progression of this syndrome^10^,^12^. This hypothesis is based on the findings of increased leukocyte count and C-reactive protein (CRP) levels in patients with ACLF compared to those of patients with cirrhosis without ACLF and their correlation with prognosis^13^. Yet bacterial infections are very common as precipitating events of ACLF, it has been suggested that the inflammatory reaction in ACLF may occur in the absence of bacterial infections, at least undetectable by current standard diagnostic methods^10^,^13^. Nonetheless, despite these suggestive findings, there is very little information on the type of inflammatory mediators that are increased in ACLF and its relationship with outcomes. Moreover, the few studies published have investigated only either single or limited number of cytokines^9^,^14^; therefore neither a complete picture about the characteristics of the inflammatory reaction nor the types of pathways involved are known. Therefore, the current study was aimed at addressing the issue of the inflammatory response and its relationship with ACLF and survival in patients with cirrhosis. A large number of cytokines was measured in patients with and without ACLF using a multiplex approach. Moreover, the results were analyzed with a principal component analysis and functional enrichment analysis to gain further insight on activated inflammatory pathways. Our findings demonstrate that the syndrome of ACLF is characterized by marked inflammatory reaction with activation of mediators of adhesion and migration of leukocytes, particularly monocytes and macrophages. Moreover, the levels of some of these cytokines are associated with prognosis, a finding that links the inflammatory reaction with outcome in ACLF.

## Results

### Baseline characteristics of patients

The current study includes 55 patients with decompensated cirrhosis, 26 with ACLF and 29 without ACLF, admitted to the Liver Unit of the Hospital Clínic in Barcelona for the management of complications of the disease. Demographic, clinical, and analytical data were collected prospectively at admission and during hospitalization. Blood samples for the measurement of cytokines were collected at the time of inclusion in the study. The characteristics of patients at time of inclusion in the study are shown in Table 1. As expected, patients with ACLF had greater frequency of ascites, hepatic encephalopathy, and shock compared to that of patients with acute decompensation of cirrhosis without ACLF. Moreover, liver function tests, Child-Pugh score and model of end-stage liver disease (MELD) score, serum creatinine, serum sodium, and mean arterial pressure were more markedly impaired in patients with ACLF than in those without ACLF. Of interest, neither the frequency of bacterial infections nor that of systemic inflammatory response syndrome (SIRS) was significantly different among groups. Leukocyte count and CRP levels were higher in patients with ACLF but the difference did not reach statistical significance.

### Cytokine levels and relationship with ACLF

**Figure 1:**
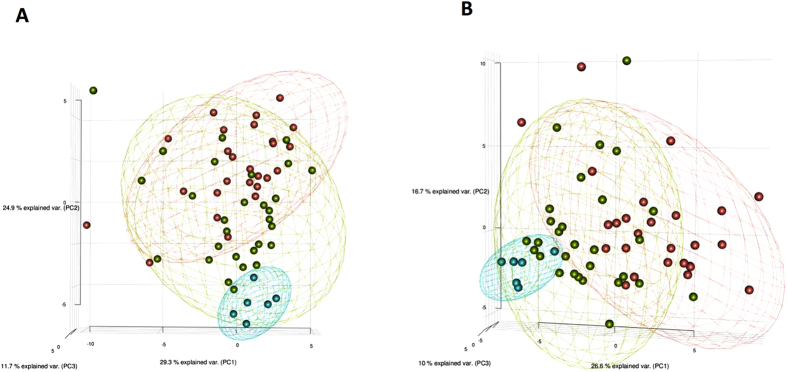
Principal Component Analysis (PCA) of all subjects included in the study with only cytokine data (Panel A) or cytokine and biochemical data (Panel B). Confidence region (95%) was indicated by an ellipsoid for each group. Each circle corresponds to one patient. Blue circles: healthy subjects; green circles: patients with acute decompensation without ACLF; Red circles: patients with ACLF.

An initial exploration of the concentrations of cytokines was performed using principal component analysis (PCA) including all cytokines with and without standard laboratory variables in all cirrhotic patients as well as in healthy subjects. Figure 1A shows a three dimensional scatter plot corresponding to the first three principal components including only cytokines. A relatively good distinction between healthy subjects, patients with acute decompensation of cirrhosis without ACLF, and patients with ACLF was observed. The group of healthy subjects was clearly separated from the other two groups. The group of patients with ACLF was scattered at the opposing end of healthy subjects, whereas patients with acute decompensation of cirrhosis without ACLF had a more heterogeneous distribution and some of them overlapped with patients with ACLF. A slightly better distinction between the 3 groups was observed when laboratory variables were added to cytokines in the principal component analysis (Fig. 1B).

Table 2 shows the levels of different cytokines in patients with and without ACLF with their respective AUCROC curves. Patients with ACLF had significantly increased levels of vascular cell adhesion molecule 1 (VCAM-1), vascular endothelial growth factor A (VEGF-A), fractalkine, macrophage inflammatory protein 1-aplha (MIP-1α), eotaxin, and interferon-inducible protein-10 (IP-10) compared to those of patients without ACLF. Levels of intercellular adhesion molecule 1 (ICAM-1) and monocyte chemoattractant protein-1 (MCP-1) were also higher in patients with ACLF but did not reach statistical significance. By contrast, levels of RANTES (Regulated on activation, normal T cell expressed and secreted), Granulocyte-macrophage colony-stimulating factor (GM-CSF), interleukin-1 beta (IL-1β), and interleukin 2 (IL-2) were significantly lower in patients with ACLF compared to those of patients without ACLF. There were no significant differences in the levels of the remaining cytokines.

**Figure 2:**
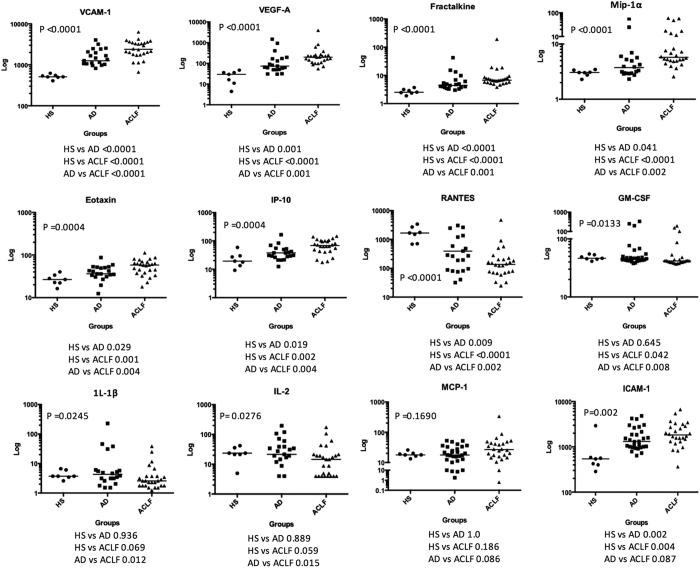
Individual values of plasma cytokines levels in healthy subjects (HS), patients with acute decompensation of cirrhosis without ACLF (AD) and patients with ACLF (ACLF). Cytokine levels are expressed in log scale.

Figure 2 shows the individual values of cytokines showing statistical significance (including the two cytokines with p values between 0.05 and 0.10) in patients with and without ACLF. A group of healthy subjects was also included for comparison. Two main messages can be derived from a close observation of this figure. First, there was overlap in the levels of most cytokines between patients with and without ACLF. Second, despite this overlap there was a clearly progressive increase (or decrease) of median cytokine levels from healthy subjects to patients without ACLF and patients with ACLF, suggesting that disease progression from decompensated cirrhosis without ACLF to ACLF is associated with significant changes in cytokine pattern. Pathway enrichment analysis using cytokines that were statistically different among groups showed that the most significant functional terms were related to migration and chemotaxis of leukocytes. Interestingly, most of the pathways were related to monocytes and macrophages (Table 3).

To assess whether the abnormal cytokine pattern found in patients with ACLF could be related to presence of bacterial infections, we next compared cytokine levels in patients with ACLF with those of the subset of patients without ACLF but with bacterial infections. The cytokines that showed statistically significant difference between the two groups of patients were the same cytokines which were significantly altered in ACLF vs no ACLF, with the only exception of VEGF-A, which showed a trend towards statistical significance (p = 0.079) (Table 4). These findings suggest that the abnormal cytokine pattern of ACLF does not appear to be related, at least for the most part, to the presence of bacterial infections.

### Cytokine levels and relationship with survival

At the end of the 3-month follow-up period, 15 patients (27%) had died, 3 (5%) had been transplanted, and the remaining 37 patients (67%) were still alive. Twelve of the 26 patients from the ACLF group died compared to only 3 of the 29 patients without ACLF (46% vs 10%, p < 0.001). The probability of survival of patients according to the presence or absence of ACLF is shown in Supplementary Figure 1. ACLF was the cause of death in all patients from the ACLF group, whereas the 3 patients from the no ACLF group died due to septic shock (two patients) and ACLF. The 3 patients transplanted belonged to the ACLF group.

**Figure 3:**
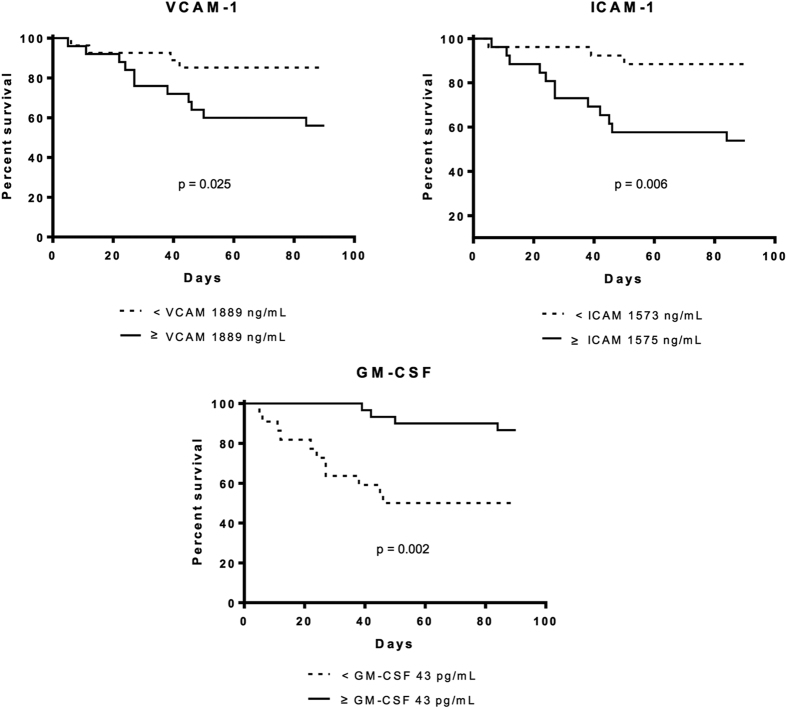
Survival probability curves of patients categorized according to median values of cytokines with prognostic value.

Several cytokines showed an association with 3-month mortality. The cytokines with strongest association with mortality (AUCROC >0.7) were VCAM-1, ICAM-1, and GM-CSF (Table 5 and Fig. 3). Increased levels of VCAM-1 and ICAM-1 were associated with increased mortality rate. By contrast, reduced levels of GM-CSF were associated with high mortality. In multivariate analysis including these 3 cytokines, only VCAM-1 was associated with independent prognostic value (Supplementary Table 1). The independent prognostic value of VCAM-1 was maintained even when individual variables known to have a powerful prognostic value, such as leukocyte count or bilirubin levels, were included in the multivariate analysis.

## Discussion

The findings of the current study indicate that ACLF syndrome is associated with an abnormal plasma cytokine profile, characterized by alterations of cytokines mainly related to chemotaxis and migration of leukocytes, particularly monocytes and macrophages. These findings confirm the existence of a marked inflammatory reaction in the setting of ACLF. The abnormal plasma cytokine profile is already present in patients with decompensated cirrhosis but is markedly enhanced in patients with ACLF. Moreover, some of the cytokines correlated with prognosis, a finding that links the inflammatory activity with disease outcome.

The landmark CANONIC study shed light on the existence and clinical relevance of systemic inflammation in decompensated cirrhosis and ACLF syndrome. Main findings of this study were: 1/ systemic inflammation, as assessed by leukocyte count and CRP levels, seems to be an important pathogenic component of the ACLF syndrome; 2/ systemic inflammation in ACLF is independent of the existence of bacterial infections; and 3/ systemic inflammation is associated with poor short-term mortality^13^. There is very little information about inflammatory cytokines in ACLF. Earlier studies showed increased serum levels of interleukin 6 (IL-6) and interleukin 10 (IL-10) in patients with ACLF compared to those of patients with stable cirrhosis, in a manner similar to that reported in patients with severe sepsis^8^. However, this study used a definition of ACLF different from the currently accepted definition. A recently published study, which investigated the expression of MERTK in immune cells showed greater serum levels of tumor necrosis factor-alpha (TNF-α), IL-6, IL-10, and interleukin 8 (IL-8) in patients with ACLF than in those without ACLF, but normal levels of Interferon gamma (IFN-ϒ), transforming growth factor beta 1 (TGF-β1), IL-1β, and interleukin 12 (IL-12)^9^, suggesting the existence of an altered cytokine profile in ACLF. The current study extends these observations by assessing a larger number of cytokines, using a multiplex approach, in a series of patients without ACLF and with well-defined ACLF. Our findings indicate that in decompensated cirrhosis without ACLF there is already an abnormal plasma cytokine profile compared to healthy subjects, which is further stressed in the setting of ACLF. The presence of abnormal plasma cytokine profile was suggested by using PCA approach and demonstrated by standard comparison of cytokine levels among groups (see Figs 1 and 2). An interesting observation of our study is that this abnormal plasma cytokine profile is not exclusively related to bacterial infections, because significant differences in cytokine levels persisted among groups when patients with ACLF were compared to the subset of patients with associated bacterial infections without ACLF.

A striking finding of our investigation was that most of the cytokines altered in patients with ACLF were related functionally with chemotaxis and migration of leukocytes, particularly monocytes and macrophages. This finding points towards an important role of monocytes/macrophages in the pathogenesis of ACLF. This observation is in keeping with previous studies showing impaired “sepsis-like” monocyte function in patients with ACLF. Wasmuth *et al*. showed that patients with ACLF have abnormal monocyte function as indicated by impaired *ex-vivo* production of TNF-α after stimulation with lipopolysaccharide (LPS) and reduced HLA-DR expression, suggesting the existence of a functional impairment of the innate immune response^8^. More recently, it has been shown that ACLF is characterized by an increased number of monocytes and macrophages that express MERTK in circulation, liver, and lymph nodes, compared to patients without ACLF and healthy controls, which correlated with inflammatory response. These MERTK-positive immune cells have an impaired response to LPS stimulation and may likely contribute to ACLF progression and infectious complications^9^. Along the same lines, our study showed that ACLF was associated with reduced levels of GM-CSF. Moreover, reduced levels of GM-CSF correlated with mortality, further suggesting the important role of the monocyte/macrophage system in determining poor outcome in cirrhosis. GM-CSF is member of a family of hematopoietic growth factors that mobilize immune cells from bone marrow and also enhances the activity of different types of leukocytes, including neutrophils and monocytes^15^,^16^. To our knowledge, there are no data on GM-CSF levels in cirrhosis and its relationship with ACLF and outcome. Interestingly, a recent study in patients and animals with experimental acute liver failure (ALF) showed that ALF is associated with reduced levels of CSF1 (also known as macrophage colony-stimulating factor, M-CSF), a growth factor that stimulates monocytes exclusively, which predicted poor outcome^17^. In the same study, it was shown that administration of CSF1 to animals with ALF improved liver regeneration. These findings are in keeping with recent studies showing that the administration of granulocyte colony-stimulating factor (G-CSF) reduces bacterial infections and improves survival in patients with decompensated cirrhosis without ACLF as well as in ACLF^18^,^19^. Although these latter studies include a small number of patients and require confirmation in larger series, overall, these data point towards an important role of impaired monocytes/macrophages function in the pathogenesis of ACLF. Further studies are needed to investigate the cause of impaired monocytes/macrophages, as well as neutrophils, function and whether colony-stimulating factors could be an effective approach to therapy of ACLF.

Another interesting observation of the current study was the markedly increased levels of VCAM-1 and ICAM-1 in patients with ACLF and their relationship with survival, so that higher levels were associated with reduced survival. VCAM-1 and ICAM-1 are molecules that are expressed in the endothelial cells, particularly in post-capillary venules, but also liver sinusoidal cells, that participate in the slow rolling, arrest and adhesion, crawling, transmigration, and diapedesis of leukocytes^20^,^21^. Because all these processes are essential initial steps in inflammation, these findings underscore the relevance of leukocyte chemotaxis and migration in ACLF and its relationship with prognosis. Similar findings of increased VCAM-1 and ICAM-1 levels have been observed in sepsis and correlate with prognosis in this condition^22^. Moreover, increased levels of ICAM-1 and VCAM-1 have been reported in patients with decompensated cirrhosis, particularly those with advanced liver failure, that correlated with prognosis^23^,^24^.

Because VCAM and ICAM are produced in endothelial cells, increased levels of these proteins have also been used as surrogate markers of endothelial dysfunction in sepsis as well as in cardiovascular diseases^25^,^26^,^27^. Therefore, our findings support the existence of endothelial dysfunction in ACLF.

It should be noted that despite that our study investigated a wide range of inflammatory mediators, there are still other potential interesting molecules (i.e., cytokines, chemokines) that could not be investigated as the number and type of measured molecules was limited to the multiplex kit selected. In this context, two recent studies have investigated the role of CXCL9 and CXCL11 in patients with cirrhosis receiving TIPS. Interestingly, results of these studies showed that increased levels of CXCL9 (monokine induced by human gamma interferon) and CXCL11 (Interferon-inducible T-cell alpha chemoattractant) were independent predictors of mortality in patients with cirrhosis receiving TIPS^28^,^29^. Therefore, it would be interesting to further investigate the role of these chemokines in the setting of ACLF in future studies.

The current study has some limitations that should be mentioned. First, the sample size is relatively low. However, patients included belong to two clearly differentiated clinical phenotypes of patients with and without ACLF, which makes the comparison between the two groups very accurate by avoiding clinical overlap. No patient without ACLF was in the process of rapid development of ACLF, which could have been a confounding factor. In fact, none of the patients without ACLF developed ACLF within one month after inclusion in the study. With respect to patients with ACLF, their characteristics were very similar to those of patients in the CANONIC study, with similar ACLF grades (50%, 31% and 19% of patients with grades I, II, and III in the current study vs 49%, 36%, and 16%, respectively, in the CANONIC study) and mortality (28-day and 90-day mortality of 27% and 46% in the current study vs 34% and 51%, respectively, in the CANONIC study). Second, plasma cytokine levels were measured exclusively at inclusion in the study; a second measurement at a later time point was not available. Therefore, assessment of changes in plasma cytokine levels according to certain outcomes, such as improvement/worsening of ACLF could not be made. However, plasma cytokines levels correlated with mortality which is the most important clinical outcome in ACLF. Finally, the use of a multiplex system allows the measurement of a large number of cytokines but is associated with some intrinsic limitations. The main limitation is that some of the cytokines included in the system could not be measured. This is probably related to cross-reading that may affect negatively the sensitivity of detection. This may explain why some cytokines that have been reported as increased in cirrhosis (i.e., TNF-α, IL-6) were not detected by the method used in the current study^6^,^30^.

In conclusion, the results of the current study show that the syndrome of ACLF is characterized by marked inflammatory reaction with activation of mediators of adhesion and migration of leukocytes, particularly monocytes and macrophages. The intensity of the inflammatory reaction correlates with prognosis.

## Methods

### Patient population and study design

The current study includes 55 patients with decompensated cirrhosis, 26 with ACLF and 29 without ACLF, admitted to the Liver Unit to the Hospital Clínic in Barcelona for the management of complications of the disease. These patients were selected from a prospective database with biobank collection that includes consecutive patients with decompensated cirrhosis admitted to hospital for treatment of an acute decompensation of the disease. Patients were randomly selected from the database from the groups with or without ACLF. Exclusion criteria were: previous kidney/liver transplantation, chronic haemodialysis before admission, hepatocellular carcinoma outside the Milan criteria or any other advanced malignancy and lack of inform consent. Causes of admission in patients without ACLF were: infection, ascites, gastrointestinal bleeding, and hepatic encephalopathy (13, 10, 4, and 2 patients, respectively). Importantly, none of the patients without ACLF developed ACLF during at least one month after sample collection. All patients with ACLF met the criteria of ACLF at admission to hospital. Causes of admission in patients with ACLF were: acute kidney injury, infection, ascites, gastrointestinal bleeding, and hepatic encephalopathy (9, 6, 4, 3, and 4 patients, respectively). A group of healthy subjects was included for comparison of plasma cytokine levels.

Demographic, clinical, and analytical data were collected prospectively at admission and during hospitalization. All complications developing during hospitalization were recorded and managed according to protocols of the Liver Unit which are based on international treatment guidelines^31^,^32^. ACLF was defined according to the CANONIC study^13^. Patients discharged from hospital were followed-up for at least 3 months.

Blood samples were collected at the time of inclusion in the study. The median time between admission to hospital and collection of samples was 1 day (0 to 3 days). Blood was centrifuged at 2,000G, at 4 °C, for 10 minutes. Plasma was stored at −80 °C until analysis. All samples were stored at the biobank as required by spanish law. All patients signed a written informed consent document and gave permission for samples to be used in the study following current national and institutional guidelines for sample storage and usage for research purposes. All the analysis and the sample collection were performed in accordance with relevant guidelines and regulations. This study was presented and approved by the Ethics Committee of Hospital Clinic registration number 2014/0577. The creation of the biobank collection was presented and approved by the Ethics Committee registration number (2011/6689).

### Multiplex cytokine assay

The following 34 cytokines and growth factors were determined with the Procarta^®^ Immunoassay Kit (Panomics, Affymetrix Inc., Santa Clara, USA): IFN-γ, fibroblast growth factor (FGF-2), interleukin-1 receptor antagonist (IL-1RA), GM-CSF, Interleukin 4 (IL-4), IL-6, IL-8, IP-10, MIP-1α, macrophage inflammatory protein 1-beta (MIP-1β), platelet-derived growth factor subunit B (PDGF-BB), TNF-α, eotaxin, IL-1β, IL-2, interleukin 5 (IL-5), interleukin 7 (IL-7), interleukin 9 (IL-9), interleukin 12 (IL-12p70), interleukin 27 (IL-27), interleukin 21 (IL-21), interleukin 15 (IL-15), MCP-1, placental growth factor-1 (PIGF-1), VEGF-A, interleukin 23 (IL-23), interleukin 17 (IL-17A), interleukin 13 (IL-13), IL-10, fractalkine, G-CSF, RANTES, VCAM-1 and ICAM-1. Briefly, 50 μL of microparticles precoated with specific antibodies were added to each well with standards or 25 μL of plasma samples and incubated for 60 minutes at room temperature in the dark. After washing the plate, 25 μL of detection antibody solution was added and the plate was incubated for 30 minutes at room temperature in the dark. A mix with streptavidin-PE solution was added to the plate for 30 minutes and then the median relative fluorescence units from the antibody reactions was measured in 120 μL of reading buffer using a Luminex 200 analyzer (Luminex, Austin, TX, USA) and the xPONENT software (v. 3.1; Luminex, Austin, TX, USA). The concentration of each analyte was calculated using five-parameter regression models. We only considered standard points with recoveries ranging from 70 to 130%. The intra-assay coefficient of variation was less than 8%. In individual samples with values below the detection limits, the lower level of detection was used in the calculation of the results. Cytokines in which the levels were below the detection limit in more than 30% of the samples (n = 14) were excluded from the analysis (the median number of samples below detection limits in this subset was 69%). These cytokines were: FGF-2, IL-1RA, IL-4, IL-8, TNF-α, IL-5, IL-9, IL-12p70, IL-27, IL-21, IL-15, PIGF-1, IL-23, and IL-17A.

### Principal component analysis

This is a technique developed for simplifying a dataset, based on orthogonal linear transformations, that converts the data to a new coordinate system such that the greatest variance by any projection of the data comes to lie on the first coordinate (first principal component), the second greatest variance on the second coordinate, and so on^33^. By this way, it may be determined whether the variation of protein abundance represents defined patterns that correlate with the already defined sample groups. We performed PCA of the integration of multiplex cytokines abundance and other biochemical data in R platform (www.r-project.org) using “stats” and “rgl” packages. An ellipsoid from the covariance matrix was drawn to show the confidence region (95%) for each of the three plotted groups.

### Statistical analysis

Categorical variables were compared with the Chi-Square test. Comparisons of parametric continuous variables between groups were made with Student’s T-test or ANOVA. Comparisons of non-parametric continuous variables between groups were made with Mann-Whitney U or Kruskal-Wallis tests. Alpha error was adjusted according to Bonferroni correction method in multiple comparisons. The area under the receiver-operating characteristic curves (AUCROC) was used to assess the relationship between each cytokine and outcomes, specifically: ACLF and 90-day transplant-free survival. Survival probability curves were calculated with the Kaplan-Meier method and compared with log-rank test. Multivariate Cox regression was performed to identify the independent factors associated with mortality. All statistical analyses were performed using SPSS 20.0 software. Results for continuous variables are expressed as median and interquartile range (IQR). Categorical variables are expressed as number and percentage. The significance level for all tests was set at 0.05 two-tailed.

### Functional enrichment analysis

Functional analysis was analyzed through the use of QIAGEN’s Ingenuity Pathway Analysis (IPA^®^, QIAGEN Redwood City, www.qiagen.com/ingenuity). The significance value associated with the functional enrichment analysis for a given dataset is a measure of the likelihood that the association between a set of focus molecules in the experiment and a given process or pathway is not due to random chance. The p-value is calculated using the right-tailed Fisher Exact Test. We selected 1.0e^−07^ as the significance threshold.

## Additional Information

**How to cite this article**: Solé, C. *et al*. Characterization of Inflammatory Response in Acute-on-Chronic Liver Failure and Relationship with Prognosis. *Sci. Rep.* **6**, 32341; doi: 10.1038/srep32341 (2016).

## Supplementary Material

Supplementary Information

## Figures and Tables

**Table 1:** Demographic, clinical, and laboratory characteristics of patients included in the study.

	All patients n = 55	Acute decompensation without ACLF n = 29	ACLF n = 26	P*
*Age (yr)*	60 (51–68)	61 (52–72)	57 (51–65)	0.235
*Male sex*	42 (76)	22 (76)	20 (77)	0.926
*Alcoholic cirrhosis*	27 (49)	13 (45)	14 (54)	0.504
*Presence of ascites*	44 (80)	19 (66)	25 (96)	0.005
*Presence of encephalopathy*	14 (26)	2 (7)	12 (46)	0.001
*Presence of bacterial infection*	31 (56)	14 (48)	17 (65)	0.201
*Presence of SIRS*	32 (58)	18 (62)	14 (54)	0.537
*Presence of shock*	5 (9)	0 (0)	5 (19)	0.013
*Serum bilirubin (mg*/*dL)*	3.1 (1.6–8.6)	2.6 (1.5–3.2)	6.2 (2.2–16)	0.009
*Serum albumin (g*/*L)*	28 (25–31)	28 (25–31)	28 (25–32)	0.695
*INR*	1.6 (1.3–2.1)	1.4 (1.2–1.6)	2.0 (1.6–2.9)	<0.0001
*Platelet count (×10*^*9*^/*L)*	76 (52–135)	114 (66–180)	60 (51–90)	0.009
*MELD score*	21 (13–29)	14 (11–18)	29 (26–33)	<0.0001
*Child-Pugh score*	10 (8–11)	9 (7–10)	11 (9–12)	<0.0001
*Serum creatinine (mg*/*dL)*	1.1 (0.7–2.1)	0.8 (0.7–1.0)	2.2 (1.5–2.8)	<0.0001
*Serum sodium (mEq*/*L)*	135 (131–137)	136 (132–139)	134 (128–136)	0.018
*Mean arterial pressure (mmHg)*	81 (71–90)	89 (82–96)	72 (62–78)	<0.0001
*Leukocyte count (×10*^*9*^/*L)*	6.8(4.5–12.2)	6.1 (4.6–10.7)	8.9 (3.9–15.3)	0.337
*Polymorphonuclear count (×10*^*9*^/*L)*	4.6 (2.5–9.7)	4.3 (2.4–8.6)	6.2 (2.8–12.6)	0.310
*C-reactive protein (mg*/*dL)*	3.2 (1.5–7.4)	2.8 (1.0–7.8)	4.9 (2.2–7.4)	0.219
*CLIF-C ACLF score*	—	38 (31–43)	51 (37–55)	<0.0001
*CLIF-C AD score*	—	49 (44–60)	68 (59–75)	<0.0001

SIRS, systemic inflammatory response syndrome; INR, international normalized ratio; MELD, model for end-stage liver disease; CLIF-C ACLF score (CLIF Consortium ACLF score), CLIF-C AD score (CLIF Consortium Acute Decompensation score). Values are expressed as numbers (%) or median and IQR (in brackets).

^*^Comparison between Acute decompensation without ACLF vs ACLF group.

**Table 2:** Comparison of plasma cytokine levels between patients with acute decompensation of cirrhosis without ALCF and patients with ACLF.

Cytokine	Acute decompensation without ACLF n = 29	ACLF n = 26	P	AUCROC (95% IC)
**VCAM-1 (ng/mL)**	**1263 (1063–2023)**	**2399 (1791–3501)**	**<0.0001**	**0.780 (0.655–0.905)**
**VEGF-A (pg/mL)**	**74 (49–169)**	**201 (112–252)**	**0.001**	**0.771 (0.644–0.897)**
**Fractalkine (pg/mL)**	**4.4 (3.6–6.4)**	**6.7 (5.5–8.2)**	**0.001**	**0.756 (0.625–0.887)**
**MIP-1α (pg/mL)**	**3.7 (3.0–5.8)**	**5.7 (4.9–12.0)**	**0.002**	**0.749 (0.615–0.882)**
**RANTES (pg/mL)**	**396 (143–1124)**	**137 (71–208)**	**0.002**	**0.739 (0.603–0.874)**
**Eotaxin (pg/mL)**	**36 (29–51)**	**58 (38–69)**	**0.004**	**0.727 (0.589–0.864)**
**IP-10 (pg/mL)**	**38 (29–54)**	**69 (43–101)**	**0.004**	**0.724 (0.580–0.868)**
**GM-CSF (pg/mL)**	**46 (43–52)**	**42 (40–45)**	**0.008**	**0.709 (0.564–0.854)**
**IL-1β (pg/mL)**	**4.3 (3.2–5.6)**	**2.6 (1.8–3.7)**	**0.012**	**0.698 (0.554–0.842)**
**IL-2 (pg/mL)**	**21 (15–36)**	**15 (4–22)**	**0.015**	**0.691 (0.546–0.836)**
**ICAM-1 (ng/mL)**	**1315 (993–2393)**	**1831 (1317–2944)**	**0.087**	**0.635 (0.485–0.784)**
**MCP-1 (pg/mL)**	**18 (12–33)**	**27 (16–45)**	**0.086**	**0.635 (0.487–0.784)**
PDGF-BB (pg/mL)	99 (37**–**245)	47 (16**–**121)	0.157	0.611 (0.459**–**0.763)
IFN-**γ** (pg/mL)	13 (9**–**28)	9 (5**–**21)	0.177	0.606 (0.452**–**0.760)
IL-6 (pg/mL)	45 (19**–**81)	59 (30**–**233)	0.175	0.607 (0.455**–**0.758)
IL-7 (pg/mL)	1.6 (1.5**–**1.7)	1.5 (1.5**–**1.7)	0.241	0.592 (0.436**–**0.748)
MIP-1β (pg/mL)	122 (95**–**160)	114 (77**–**160)	0.273	0.586 (0.432**–**0.740)
IL-10 (pg/mL)	2.1 (0.7**–**4.4)	1.1 (0.08**–**5.0)	0.300	0.582 (0.425**–**0.738)
G-CSF (pg/mL)	36 (22**–**65)	49 (22**–**83)	0.680	0.532 (0.376**–**0.689)
IL-13 (pg/mL)	4.8 (4.5**–**7.8)	4.8 (4.5**–**5.4)	0.495	0.446 (0.293**–**0.599)

AUCROC, area under the receiver-operating characteristic curves.

Values are expressed as median and IQR (in brackets).

**Table 3:** Pathway enrichment analysis revealing specific immune inflammatory response pathways involved in acute-on chronic liver failure.

Enriched processes/pathways	Num	Cytokines involved Types of cytokines	P
Cell movement of monocytes	9	Eotaxin, MIP-1α, RANTES, GM-CSF, Fractalkine, IP-10, IL1-β,IL-2,VCAM-1	8.44e^−20^
Leukocyte migration	10	Eotaxin, MIP-1α, RANTES, GM-CSF, Fractalkine, IP-10, IL-1β, IL-2, VCAM-1,VEGF-A	1.82e^−18^
Migration of phagocytes	8	MIP-1α, RANTES, GM-CSF, Fractalkine, IP-10, IL-1β, IL-2, VCAM-1	3.93e^−17^
Chemotaxis of mononuclear leukocytes	8	Eotaxin, MIP-1α, RANTES, GM-CSF, Fractalkine, IP-10, IL-1β, IL-2	1.25e^−16^
Chemotaxis of monocytes	7	Eotaxin, MIP-1α, RANTES, GM-CSF, Fractalkine, IP-10, IL-1β	1.45e^−15^
Migration of monocytes	6	MIP-1α, RANTES, GM-CSF, Fractalkine, IL-2,VCAM-1	7.58e^−14^
Migration of mononuclear leukocytes	7	MIP-1α, RANTES, GM-CSF, Fractalkine, IP-10, IL-2, VCAM-1	3.45e^−13^
Cell movement of natural killer cells	5	MIP-1α, RANTES, Fractalkine, IP-10, IL-2	5.88e^−13^
Activation of macrophages	5	MIP-1α, RANTES, GM-CSF, IL-1β, IL-2	1.97e^−12^
Transmigration of phagocytes	5	MIP-1α, RANTES, GM-CSF, IL-1β, VCAM-1	9.08e^−12^
Binding of professional phagocytic cells	5	Eotaxin, MIP-1α, RANTES, IP-10, VCAM-1	2.62e^−11^
Neovascularization	4	GM-CSF, IL-1β, IL-2, VEGF-A	4.28e^−11^
Necrosis	9	Eotaxin, MIP-1α, RANTES, GM-CSF, Fractalkine, IL-1β, IL-2,VCAM-1, VEGF-A	8.39e^−11^
Chronic inflammatory disorder	9	Eotaxin, MIP-1α, RANTES, GM-CSF, IP-10, IL-1β, IL-2, VCAM-1, VEGF-A	9.40e^−11^
Cell viability	6	GM-CSF, IP-10, IL-1β, IL-2, VCAM-1, VEGF-A	3.46e^−8^

Only the most significant enriched biological processes and pathways based on the p value are shown in the table. The number of cytokines (Num) and types of cytokines overlapping with the significant pathways are also shown.

**Table 4:** Comparison of plasma cytokine levels between patients with acute decompensation of cirrhosis without ACLF with associated bacterial infections and patients with ACLF.

Cytokine	Acute decompensation without ACLF and with bacterial infection n = 14	ACLF n = 26	P
**VCAM-1 (ng/mL)**	**1665 (114–2498)**	**2399 (1791–3501)**	**0.033**
**VEGF-A (pg/mL)**	**111(60–249)**	**201 (112–252)**	**0.079**
**Fractalkine (pg/mL)**	**4.4 (3.7–10.7)**	**6.7 (5.5–8.2)**	**0.040**
**MIP-1α (pg/mL)**	**3.9 (2.9–5.7)**	**5.7 (4.9–11.9)**	**0.008**
**RANTES (pg/mL)**	**439 (166–1628)**	**137 (71–208)**	**0.020**
**Eotaxin (pg/mL)**	**35 (30–50)**	**58 (38–69)**	**0.005**
**IP-10 (pg/mL)**	**34 (26–60)**	**69 (43–101)**	**0.023**
**GM-CSF (pg/mL)**	**46 (42–53)**	**42 (40–45)**	**0.025**
**IL-1β (pg/mL)**	**4.2 (3.2–5.9)**	**2.6 (1.8–3.7)**	**0.020**
**IL-2 (pg/mL)**	**29 (18–39)**	**15 (4.0–21)**	**0.017**
ICAM-1 (ng/mL)	1422 (1001**–**2725)	1831 (1317**–**2944)	0.207
MCP-1 (pg/mL)	22 (11**–**33)	27 (16**–**45)	0.133
PDGF-BB (pg/mL)	101 (27**–**303)	47 (16**–**121)	0.233
IFN-γ (pg/mL)	14 (10**–**29)	9.1 (5.3**–**21)	0.305
IL-6 (pg/mL)	39 (19**–**107)	59 (30**–**233)	0.301
IL-7 (pg/mL)	1.6 (1.5**–**1.8)	1.5 (1.5**–**1.7)	0.255
MIP-1β (pg/mL)	122 (91**–**159)	114 (77**–**161)	0.514
IL-10 (pg/mL)	2.4 (0.7**–**4.9)	1.1 (0.08**–**5.0)	0.282
G-CSF (pg/mL)	36 (22**–**50)	49 (22**–**83)	0.487
IL-13 (pg/mL)	4.9 (4.5**–**7.2)	4.8 (4.5**–**5.4)	0.660

Values are expressed as median and IQR (in brakets).

**Table 5:** Comparison of plasma cytokine levels in patients included in the study categorized according to 3-month survival.

Variables	Alive (n = 37)	Dead (n = 15)	P	Mortality AUCROC (95% IC)
**VCAM-1 (ng/mL)**	**1263 (1072–2244)**	**2942 (1803–3471)**	**0.001**	**0.807 (0.690–0.925)**
**ICAM-1 (ng/mL)**	**1315 (978–1897)**	**2633 (1704–3178)**	**0.001**	**0.791 (0.667–0.915)**
**GM-CSF (pg/mL)**	**46 (42–52)**	**41 (40–43)**	**0.013**	**0.721 (0.557–0.884)**
**MIP-1α (pg/mL)**	**4.2 (3.2–6.2)**	**6.0 (4.9–17.01)**	**0.028**	**0.695 (0.540–0.851)**
**IP-10 (pg/mL)**	**41 (29–72)**	**64 (49–106)**	**0.040**	**0.683 (0.523–0.842)**
**RANTES (pg/mL)**	**287 (85–895)**	**125 (71–280)**	**0.077**	**0.658 (0.499–0.816)**
**IL-2 (pg/mL)**	**19 (14.6–36)**	**15 (4.0–27)**	**0.077**	**0.658 (0.479–0.836)**
IL-1β (pg/mL)	4.0 (2.6**–**5.9)	3.2 (1.8**–**3.7)	0.108	0.643 (0.483**–**0.804)
MPC-1 (pg/mL)	17.8 (12.4**–**33.9)	27.6 (17.5**–**48.4)	0.138	0.632 (0.458**–**0.807)
IL-7 (pg/mL)	1.6 (1.5**–**1.8)	1.5 (1.5**–**1.6)	0.146	0.630 (0.471**–**0.789)
PDGF-BB (pg/mL)	84 (38**–**272)	46 (16**–**93)	0.183	0.619 (0.456**–**0.782)
IL-13 (pg/mL)	5.0 (4.5**–**6.3)	4.6 (4.5**–**5.1)	0.229	0.607 (0.450**–**0.764)
Fractalkine (pg/mL)	5.4 (3.8**–**7.3)	6.3 (5.1**–**7.6)	0.233	0.606 (0.456**–**0.757)
Eotaxin (pg/mL)	43 (33**–**58)	44 (32**–**81)	0.486	0.562 (0.367**–**0.757)
IL-10 (pg/mL)	2.1 (0.5**–**4.4)	1.3 (0.1**–**4.8)	0.525	0.557 (0.382**–**0.731)
VEGF-A (pg/mL)	140 (55**–**221)	163 (87**–**224)	0.538	0.555 (0.391**–**0.719)
MIP-1β (pg/mL)	122 (91**–**160)	116 (85**–**159)	0.754	0.528 (0.355**–**0.701)
G-CSF (pg/mL)	38 (25**–**69)	42 (15**–**83)	1.000	0.500 (0.312**–**0.688)
IL-6 (pg/mL)	47 (21**–**100)	45 (25**–**117)	0.976	0.497 (0.323**–**0.672)
IFN-γ (pg/mL)	11 (8**–**28)	15 (4.7**–**18)	0.777	0.475 (0.287**–**0.662)

AUCROC, area under the receiver-operating characteristic curves.

Values are expressed as median and IQR (in brackets).

The 3 patients transplanted during the 3-month follow-up period were excluded from the analysis.
