# Supplementary material for: Characterization of Inflammatory Response in Acute-on-Chronic Liver Failure and Relationship with Prognosis
Source: Sci Rep. 2016 Aug 31;6:32341. doi: 10.1038/srep32341 (PMC5006032; doi:10.1038/srep32341)

CHARACTERIZATION OF INFLAMMATORY RESPONSE IN ACUTE-ON-CHRONIC LIVER FAILURE AND RELATIONSHIP WITH PROGNOSIS

Cristina Solé[1,2,3], Elsa Solà[1,2,3]*, Manuel Morales-Ruiz[2,3,4], Guerau Fernàndez[5], Patricia Huelin[1,2,3], Isabel Graupera[1,2,3], Rebeca Moreira[1,2,3], Gloria de Prada[1,2,3], Xavi Ariza[1,2,3], Elisa Pose[1,2,3], Núria Fabrellas[2,6], Susana G. Kalko[5], Wladimiro Jiménez[2,3,4], Pere Ginès[1,2,3].

1. Liver Unit, Hospital Clínic de Barcelona, University of Barcelona, Barcelona, Spain.
2. Institut d'Investigacions Biomèdiques August Pi i Sunyer (IDIBAPS), Barcelona, Spain.
3. Centro de Investigación Biomédica en Red de Enfermedades Hepáticas y Digestivas (CIBEReHD), Barcelona, Spain.
4. Biochemistry and Molecular Genetics Department, Hospital Clínic de Barcelona, Barcelona, Spain.
5. Bioinformatics Core Facility, IDIBAPS-CEK, Hospital Clínic, University de Barcelona, Spain.
6. School Of Nursing, University of Barcelona, Barcelona, Spain.

*Address for correspondence:

Elsa Solà MD, PhD
Liver Unit
Hospital Clinic de Barcelona
Barcelona
Spain
Email: esola@clinic.cat

SUPPLEMENTARY TABLE

Supplementary table 1. Multivariate analysis of factors associated with mortality at 3-month.

Model 1.

Variables	*HR*	*95% CI*	*P*
**VCAM-1**	3.3	1.1-9.2	0.026

*Variables included in the model (log transformed): VCAM-1, ICAM-1, and GM-CSF.

Model 2.

Variables	*HR*	*95% CI*	*P*
**VCAM-1**	4.4	1.5-12.8	0.007
**Leukocyte count**	2.3	1.1-5.1	0.032

* Variables included in the model (log transformed): VCAM-1, leukocyte count, and bilirubin.

SUPPLEMENTARY FIGURE

Supplementary Figure 1. Survival probability curves of patients categorized according to the presence of ACLF.

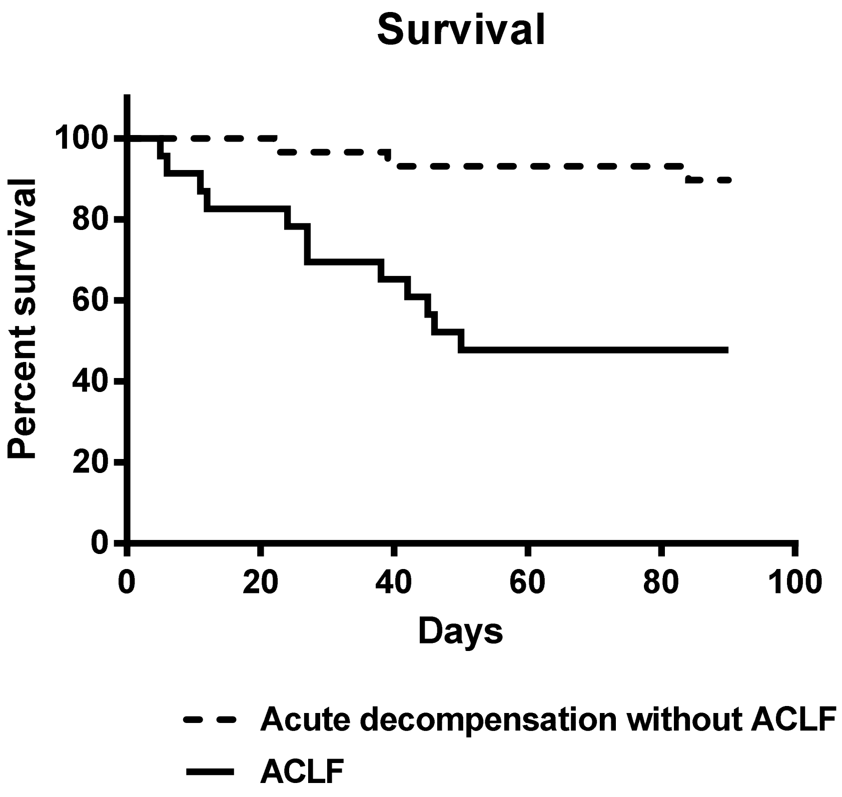

Supplement: Supplementary Information [file srep32341-s1.pdf]
